# TBC-HRL: A Bio-Inspired Framework for Stable and Interpretable Hierarchical Reinforcement Learning

**DOI:** 10.3390/biomimetics10110715

**Published:** 2025-10-22

**Authors:** Zepei Li, Yuhan Shan, Hongwei Mo

**Affiliations:** 1 College of Intelligent Systems Science and Engineering, Harbin Engineering University, No.145 Nantong Street, Harbin 150001, China; 221040013@hrbeu.edu.cn; 2 China Academy of Electronics and Information Technology, Shijingshan District, Beijing 100041, China; shanyuhan@cetc.com.cn

**Keywords:** hierarchical reinforcement learning, bionic neural circuits, timed subgoal scheduling, liquid time-constant network, neurodynamic control, robot learning, autonomous robots, intelligent control systems

## Abstract

Hierarchical Reinforcement Learning (HRL) is effective for long-horizon and sparse-reward tasks by decomposing complex decision processes, but its real-world application remains limited due to instability between levels, inefficient subgoal scheduling, delayed responses, and poor interpretability. To address these challenges, we propose Timed and Bionic Circuit Hierarchical Reinforcement Learning (TBC-HRL), a biologically inspired framework that integrates two mechanisms. First, a timed subgoal scheduling strategy assigns a fixed execution duration τ to each subgoal, mimicking rhythmic action patterns in animal behavior to improve inter-level coordination and maintain goal consistency. Second, a Neuro-Dynamic Bionic Circuit Network (NDBCNet), inspired by the neural circuitry of C. elegans, replaces conventional fully connected networks in the low-level controller. Featuring sparse connectivity, continuous-time dynamics, and adaptive responses, NDBCNet models temporal dependencies more effectively while offering improved interpretability and reduced computational overhead, making it suitable for resource-constrained platforms. Experiments across six dynamic and complex simulated tasks show that TBC-HRL consistently improves policy stability, action precision, and adaptability compared with traditional HRL, demonstrating the practical value and future potential of biologically inspired structures in intelligent control systems.

## 1. Introduction

Reinforcement Learning (RL) has demonstrated strong potential in solving complex control tasks and has found wide applications in areas such as robot navigation, robotic manipulation, and autonomous driving [1,2,3,4]. Among various RL architectures, Hierarchical Reinforcement Learning (HRL) is particularly effective with its “high-level decision–low-level execution” structure, which improves sample efficiency and enhances policy generalization in long-horizon tasks. By decomposing complex problems into subgoals, HRL alleviates sparse-reward challenges and supports modular policy design [5,6,7,8]. Nevertheless, despite these theoretical advantages, HRL still faces critical limitations in real-world deployment, including unstable inter-level coordination, the absence of rhythmic subgoal scheduling, insufficient responsiveness at the low level, and high computational costs [9,10].

These challenges can be summarized into three aspects. First, inter-level coordination is often unstable: since high-level policies depend on low-level feedback, policy bias may occur before the low-level converges, leading to slow learning or oscillation. Frequent subgoal switching further introduces scheduling imbalance, reducing behavioral consistency and efficiency [11,12]. Second, low-level controllers are often insufficiently responsive. Conventional fully connected networks adapt slowly to abrupt environmental changes, while reactive policies lack the memory capacity to integrate historical information, making them inadequate for modeling temporal dependencies such as velocity variations, obstacle distributions, and energy consumption patterns [13]. Third, current policy networks are limited in both interpretability and computational efficiency. Most are implemented as “black-box” models whose decision origins are difficult to trace, raising safety concerns; moreover, large parameter sizes and significant overhead hinder HRL deployment on embedded or resource-constrained platforms [14].

To address these issues, recent studies have drawn inspiration from biological neural systems and behavioral mechanisms [15,16]. In nature, organisms exhibit strong adaptability, robustness, and energy efficiency, with neural regulatory systems forming clearly defined hierarchical structures for perception, decision making, and control [17]. For example, insects can achieve stable path planning and goal tracking with minimal neural structures; C. elegans generate complex motor behaviors with only a small number of interconnected neurons [18]; and vertebrates rely on multi-level coordination between the cerebral cortex and brainstem to achieve motor control [19]. These cases suggest that hierarchical decision structures, synaptic modulation, and temporally regulated strategies are essential for stable behavior generation. In particular, many animals reevaluate environmental feedback only after completing a behavioral unit, thereby improving efficiency and continuity. This “goal maintenance–feedback evaluation–periodic updating” mechanism inspires efficient subgoal scheduling and stable learning. Furthermore, biological neural systems exhibit structural sparsity, rapid responsiveness, and strong information retention, providing new directions for designing interpretable and efficient network architectures in control systems.

Inspired by these biological mechanisms, we propose TBC-HRL, a bio-inspired framework designed to overcome the limitations of traditional HRL in scheduling stability, control responsiveness, and computational efficiency. The framework integrates two core components.

First, the Timed Subgoal Scheduling mechanism assigns a fixed execution duration (τ) to each subgoal, drawing on the temporal persistence principle in biological behaviors. This reduces disruptions from frequent subgoal switching, improves inter-level coordination, and stabilizes low-level learning and responsiveness.

Second, the Neuro-Dynamic Bionic Circuit Network (NDBCNet), inspired by the neural circuitry of C. elegans, replaces conventional fully connected networks in the low-level policy. With sparse connectivity, continuous-time dynamics, and adaptive responses, NDBCNet effectively models temporal dependencies, strengthens behavioral regulation, and enhances interpretability. Its compact parameterization further reduces computational overhead while maintaining robust performance.

By combining these mechanisms, TBC-HRL achieves more stable, responsive, and interpretable control, highlighting the practical value of bio-inspired structures in reinforcement learning.

The main contributions of this work are as follows:

- We introduce TBC-HRL, a hierarchical reinforcement learning framework that integrates timed subgoal scheduling with a biologically inspired neural circuit model (NDBCNet), addressing core challenges of unstable coordination, delayed response, and limited interpretability in HRL.
- We evaluate TBC-HRL across six simulated robot tasks with sparse rewards and long horizons, demonstrating consistent improvements in sample efficiency, subgoal stability, and policy generalization compared to standard HRL methods.
- We analyze NDBCNet in detail and show how structural sparsity, temporal dynamics, and adaptive responses contribute to robust and interpretable low-level control in complex environments.

## 2. Related Work

### 2.1. Limitations of Hierarchical Reinforcement Learning in Real-World Tasks

HRL achieves high sample efficiency and strong policy generalization in complex control tasks by decomposing policies into different levels. High-level policies generate subgoals while low-level policies execute them, thereby alleviating the sparse reward problem in long-horizon tasks. Representative approaches include Option-Critic, FeUdal Networks, HIRO, and HiTS [20,21,22,23]. Nevertheless, HRL still encounters several critical challenges in real-world scenarios.

First, high-level policies rely heavily on feedback from low-level policies, and the instability of low-level learning during early training stages often prevents high-level convergence, leading to non-stationary updates. Second, the absence of rhythmicity and temporal coordination in subgoal scheduling frequently causes excessive switching, which disrupts low-level learning efficiency. Third, most existing HRL frameworks employ fully connected neural networks as policy approximators, which are structurally complex, slow to respond, and limited in interpretability, making them unsuitable for real-time, energy-efficient, and stable control applications.

Thus, there is an urgent need for hierarchical architectures that incorporate rhythm-aware scheduling, lightweight network structures, and bio-inspired characteristics to enhance inter-level coordination stability and reduce response delays.

### 2.2. Subgoal Scheduling and Temporal Abstraction Mechanisms

In HRL, the generation and scheduling of subgoals by the high-level policy are crucial for overall performance and stability. Various approaches have been proposed to improve scheduling rationality and adaptability. For instance, HIRO employs fixed-interval scheduling, which is simple but lacks environmental adaptability. FeUdal Networks and SNN-HRL [24] introduce state-triggered mechanisms that enhance sensory responsiveness but may cause policy oscillation. HiTS adopts a learnable switching strategy that autonomously determines subgoal switching points; however, without explicit temporal constraints, it can result in frequent switching and fragmented learning in low-level policies.

Furthermore, as low-level policies continuously evolve during training, the high-level policy operates in a non-stationary SMDP, where state transitions change dynamically, hindering convergence and reducing sample efficiency. Although techniques such as hindsight relabeling and adjacency loss have been proposed to mitigate this issue, their effectiveness remains constrained by policy stability and the complexity of the subgoal space.

It is worth noting that most existing methods neglect the temporal continuity and behavioral rhythmicity of subgoal execution. In real-world tasks, the absence of these properties often produces discrete and unstable scheduling signals, undermining coordination between high and low levels. In contrast, biological organisms frequently achieve behavioral stability through rhythmic and sustained action sequences—such as insect foraging or animal motor control—which inspire the incorporation of explicit temporal constraints into high-level scheduling to improve stability and execution efficiency.

Unlike prior work that primarily focuses on when subgoals should be switched (i.e., scheduling time), our framework emphasizes the explicit modeling of subgoal duration τ. By assigning a fixed execution horizon to each subgoal, the high-level controller enforces rhythmic temporal abstraction, reduces excessive switching, and stabilizes inter-level coordination. This distinction highlights our contribution: shifting from timing-based subgoal triggers toward duration-aware scheduling, thereby enhancing long-horizon credit assignment and improving overall policy stability.

### 2.3. Applications of Bio-Inspired Neural Network Structures in Intelligent Control

In recent years, inspired by biological nervous systems, researchers have proposed a range of bio-inspired neural network architectures with strong temporal modeling and dynamic regulation capabilities. These include Spiking Neural Networks (SNNs) [25], Liquid Time-Constant Networks (LTC) [26], and Neural Circuit Policies (NCPs) [18]. By leveraging sparse connectivity and dynamic neuron state evolution, these models emulate membrane potential dynamics and demonstrate strong abilities in time-series modeling, adaptive control, and interpretability. They have shown broad potential in tasks such as robotic control, motion prediction, and sequential decision-making [27,28,29].

Compared with conventional fully connected neural networks, bio-inspired structures generally require fewer parameters, achieve higher computational efficiency, and provide stronger robustness, making them particularly suitable for resource-constrained or real-time control scenarios. However, most existing studies focus on applying bio-inspired neural networks within single-layer policy frameworks. Their integration into hierarchical control architectures remains underexplored, leaving ample opportunities for future development.

## 3. Background

### 3.1. MDP and SMDP

RL problems are typically modeled as a Markov Decision Process (MDP), defined by a state space S, an action space A, transition dynamics $P(s^{\prime }|s,a)$, a reward function R(s,a), and a discount factor γ∈[0,1]. An agent interacts with the environment according to a policy π(a|s) to maximize the expected discounted return:

(1) $$J\left(\pi \right)=E_{\pi }\left[\sum_{t=0}^{\infty }\gamma^{t}r_{t}\right].$$

While MDPs are suitable for many tasks, they struggle in long-horizon and sparse-reward problems due to single-step decision granularity. To address this, HRL extends to a Semi-Markov Decision Process (SMDP), where high-level policies output temporally abstract actions (subgoals) executed by low-level policies for τ steps. This abstraction improves exploration but introduces non-stationary dynamics, since the high-level policy depends on the evolving low-level policy, and stability is sensitive to the choice of τ.

### 3.2. Subgoal-Based HRL

In subgoal-based HRL, a high-level controller generates subgoals $g_{t}\in G$, while a low-level policy executes primitive actions to achieve them. The scheduling of subgoals is thus critical for overall performance. Existing approaches adopt different strategies: HIRO [22] uses fixed temporal intervals, offering simplicity but poor adaptability; FeUdal Networks and SNN-HRL [24] rely on state-triggered updates, which improve reactivity but may induce oscillations; HiTS [23] learns switching points adaptively, but without explicit temporal constraints, frequent switching and fragmented behaviors may occur.

Overall, current methods lack rhythm-aware scheduling and adaptive temporal abstraction, which limits stability and efficiency in real-world deployments. These issues motivate the development of mechanisms that combine explicit temporal coordination with robust hierarchical control, as pursued in this work.

## 4. Method

### 4.1. Overall Architecture: TBC-HRL

**Figure 1:**
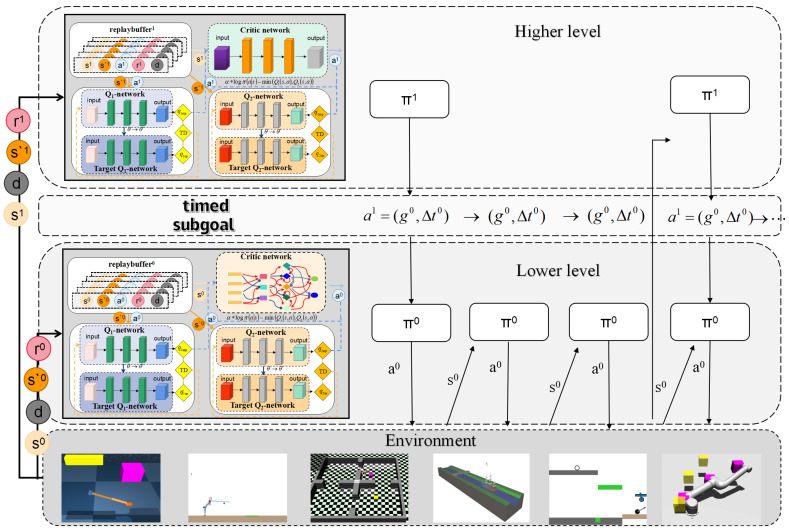
Timed and bionic circuit hierarchical reinforcement learning.

We propose TBC-HRL, a two-level hierarchical reinforcement learning framework that integrates a high-level SAC policy with a low-level SAC policy parameterized by an NDBCNet. The overall structure of the proposed framework is illustrated in Figure 1, which provides a system-level overview of the interaction between the high-level controller, the low-level controller, and the environment. The high-level controller $\pi_{1}$ operates at a coarse temporal scale and generates a spatial subgoal $g^{0}$ together with a time budget $\Delta t^{0}$ through the timed subgoal scheduling mechanism, ensuring temporal coordination between levels. The low-level controller $\pi_{0}$ interacts with the environment at a fine timescale, executing actions conditioned on the subgoal and its budget. Both levels maintain separate replay buffers ($D_{H},D_{L}$) and Q-function critics for SAC updates, enabling decoupled yet coordinated optimization. This design enhances stability, sample efficiency, and generalization in long-horizon and sparse-reward tasks.

At a high-level decision step *t*, the policy outputs a joint action:

(2) $$a^{1}=\left(g^{0},\Delta t^{0}\right),$$

where $g^{0}$ denotes the spatial subgoal (e.g., target position or state vector) and $\Delta t^{0}$ specifies the execution horizon in low-level timesteps. The high-level reward $r_{t}^{H}$ is computed from task progress and updated less frequently, typically once every $\Delta t^{0}$ low-level steps.

We treat the time budget Δt as a temporal abstraction that low-pass filters high-level switching: too small Δt induces frequent re-synchronization and cross-level non-stationarity, whereas too large Δt yields sluggish reactions to exogenous events. In practice, we adopt a simple, reproducible rule that scales Δt with the typical subgoal reachability:

(3) $$\Delta t\;=\;\mathrm{clip}\;\left(\left\lceil \kappa \;{\hat{H}}_{\mathrm{reach}}\right\rceil ,\;\Delta t_{\min},\;\Delta t_{\max}\right),$$

where ${\hat{H}}_{\mathrm{reach}}$ is the estimated geodesic steps (or a model-based proxy) to reach the subgoal under nominal dynamics, κ∈[0.6,1.2] is a dimensionless scaling factor, and $\Delta t_{\min},\Delta t_{\max}$ are task-level safety bounds. This choice preserves the rhythmic execution that improves temporal credit assignment and inter-level coordination in timing-critical settings, while remaining simple and compute-efficient.

We use a single symbol Δt for subgoal duration throughout the paper. At each high-level decision step *k*, the high-level policy jointly predicts $\left(g_{k},\Delta t_{k}\right)$ once and then holds $\Delta t_{k}$ fixed as the execution window; no re-prediction occurs inside the window. Unless otherwise stated, Δt is treated as a continuous value clipped to a feasible range $\left[\Delta t_{\min},\Delta t_{\max}\right]$; in strictly discrete-time environments we round it to the nearest integer number of steps. The low level receives the remaining-time input and we normalize it as

(4) $${\tilde{\Delta t}}_{\mathrm{rem}}\;=\;\mathrm{clip}\;\left(\frac{\Delta t_{\mathrm{rem}}}{\bar{\Delta t}},\;0,\;1\right).$$

where $\bar{\Delta t}$ is a per-environment reference scale reported in the appendix.

For the high level, the SMDP target explicitly couples the chosen duration with discounting:

(5) $$y_{H}\;=\;R_{k}^{\left(\Delta t\right)}\;+\;\gamma^{\Delta t}\;V_{H}\;\left(s_{k+\Delta t}\right).$$

(6) $$R_{k}^{\left(\Delta t\right)}\;=\;\sum_{j=0}^{\Delta t-1}\gamma^{\;j}\;r_{k+j}.$$

In SAC form,

(7) $$V_{H}\left(s\right)\;=\;E_{a∼\pi_{H}\left(\cdot \;|\;s\right)}\;\left[\min_{i=1,2}Q_{H}^{\left(i\right)}\left(s,a\right)\;-\;\alpha_{H}\log\pi_{H}\left(a\;|\;s\right)\right].$$

Inside the window the low level uses standard per-step SAC targets conditioned on $\left(g,{\tilde{\Delta t}}_{\mathrm{rem}}\right)$ (no SMDP skip).

At each low-level timestep $t^{\prime }$, the policy $\pi_{0}$ receives the local state $s_{t^{\prime }}$, the subgoal $g^{0}$, and its time budget $\Delta t^{0}$, and outputs a control action:

(8) $$a^{0}∼\pi_{0}\left(a^{0}|s_{t^{\prime }},g^{0},\Delta t^{0}\right),$$

which is directly applied to the actuators (e.g., joint velocities or thrust vectors). The low-level reward $r_{t^{\prime }}^{L}$ measures the degree of subgoal completion and is updated at every step.

The dynamics of NDBCNet are modeled in continuous time:

(9) $$\dot{h}\left(t\right)=f_{\theta }\left(h\left(t\right),x\left(t\right)\right),\;h_{t+1}=h_{t}+\delta t\cdot f_{\theta }\left(h_{t},x_{t}\right),$$

where $h_{t}$ is the hidden state, $x_{t}$ the input, and δt the integration step size. This design captures temporal dependencies, enhances interpretability, and significantly reduces parameter complexity.

The temporal relationship between levels is as follows:

(10) $$t^{\prime }\in \left[\tau_{k},\tau_{k}+\Delta t^{0}\right),$$

where $\tau_{k}$ is the starting low-level timestep for the *k*-th high-level decision. The low-level policy executes the subgoal until the budget expires or the subgoal is completed earlier.

The overall objective is to maximize the joint expected return:

(11) $$J\left(\pi_{1},\pi_{0}\right)=E\;\left[\sum_{t}\gamma_{H}^{t}r_{t}^{H}+\sum_{t^{\prime }}\gamma_{L}^{t^{\prime }}r_{t^{\prime }}^{L}\right],$$

where $\gamma_{H}$ and $\gamma_{L}$ are the discount factors for the high- and low-level controllers. Both policies are optimized following the standard SAC objective with entropy regularization.

Unlike HIRO, which uses fixed subgoal update intervals, and HiTS, which infers duration implicitly via learned switch points, our timed subgoal scheduling (TS) treats time as an explicit control resource: the high level jointly outputs a subgoal *g* and a time budget Δt, and execution at the low level is constrained to this window, yielding a rhythm-aware structure. This design (i) injects $\gamma^{\Delta t}$ explicitly into the SMDP targets and replay sampling, stabilizing inter-level credit assignment and reducing high-level chattering; and (ii) conditions the low-level policy on the remaining budget $\Delta t_{\mathrm{rem}}$ at each step, enabling time-aware action allocation and energy–accuracy trade-offs. Rather than merely “predicting a duration,” TS couples the chain of “duration decision → discount propagation → low-level control → replay relabeling (including HGR)” end-to-end in the optimization pipeline, improving rhythmic stability, sample reuse, and reproducibility. The feasible range of Δt is specified via practical upper/lower bounds and priors (implementation details and appendix), ensuring robustness during training and deployment.

### 4.2. High-Level Policy Generation

In the TBC-HRL framework, the high-level policy generates a spatial subgoal $g^{0}$ together with its execution duration $\Delta t^{0}$, forming the high-level action $a^{1}$ that guides the low-level controller over the interval $\left[t,t+\Delta t^{0}\right]$. The controller is built on the Soft Actor-Critic (SAC) framework, while incorporating explicit temporal abstraction and hindsight relabeling to enhance stability and efficiency.

The high-level policy is modeled as a joint distribution:

(12) $$\left(g^{0},\Delta t^{0}\right)∼\pi_{1}\left(g,\Delta t|s_{t}\right),$$

where $g^{0}\in G$ is the selected subgoal and $\Delta t^{0}\in R_{+}$ denotes its execution horizon. Unlike conventional HRL that only outputs subgoals, our design explicitly incorporates temporal constraints to improve coordination and rhythmic scheduling.

To enable stable temporal abstraction, the execution duration $\Delta t^{0}$ is optimized using a regression loss:

(13) $$L_{\Delta t}=E_{s_{t}}\;\left[{\left(\Delta t^{0}-\Delta t^{*}\right)}^{2}\right],$$

where $\Delta t^{*}$ is a pseudo-label obtained from execution feedback or trajectory statistics. This mechanism prevents frequent goal switching and ensures consistent subgoal execution.

To mitigate non-stationarity in high-level transitions and improve sample efficiency, we adopt hindsight relabeling with unified symbols. At time *t*, the high-level policy $\pi_{0}$ outputs a subgoal $g^{0}$ and a duration $\Delta t^{0}$; $\Delta t^{0}\in N_{+}$ (or $R_{+}$ in continuous-time variants) is predicted once and then held fixed during execution, with bounds $\Delta t^{0}\in \left[\Delta t_{\min},\Delta t_{\max}\right]$. States provided to the critics (for both $\pi_{0}$ and the low-level policy $\pi_{1}$) are normalized per environment, and the remaining-time feature is min–max scaled as ${\tilde{\tau }}_{k}=\frac{\Delta t^{0}-k}{\Delta t_{\max}}\in \left[0,1\right]$.

When the original subgoal is not achieved, it is relabeled by the final state reached at the end of the option, and success for the relabeled transition is determined with a fixed radius $\varepsilon_{\mathrm{rel}}=0.05$ (on the normalized state space):

(14) $${\hat{g}}^{0}=s_{t+\Delta t^{0}},\;{\hat{r}}_{t}^{H}=\left\{\begin{array}{cc} 1, & \mathrm{if}\;∥\phi \left(s_{t+\Delta t^{0}}\right)-\phi \left({\hat{g}}^{0}\right){∥}_{2}\leq \varepsilon_{\mathrm{rel}},\\ 0, & \mathrm{otherwise}. \end{array}\right.$$

Here ϕ(·) selects the goal-relevant coordinates (identity in our default setting). This relabeling densifies reward signals and enables efficient training even when the low-level policy $\pi_{1}$ is still being optimized.

The high-level module integrates SAC-based optimization, explicit temporal abstraction, and hindsight relabeling, achieving efficient, rhythmic, and biologically inspired subgoal scheduling.

### 4.3. Neuro-Dynamic Bionic Control Network

To enhance the responsiveness and control accuracy of the low-level policy, we introduce the NDBCNet, a biologically inspired neural architecture motivated by the compact and efficient nervous system of C. elegans. Unlike conventional fully connected networks that rely on discrete layers and fixed-step updates, NDBCNet adopts sparse connectivity, continuous-time dynamics, and excitatory/inhibitory regulation, enabling fine-grained temporal modeling and robust control in reinforcement learning.

**Figure 2:**
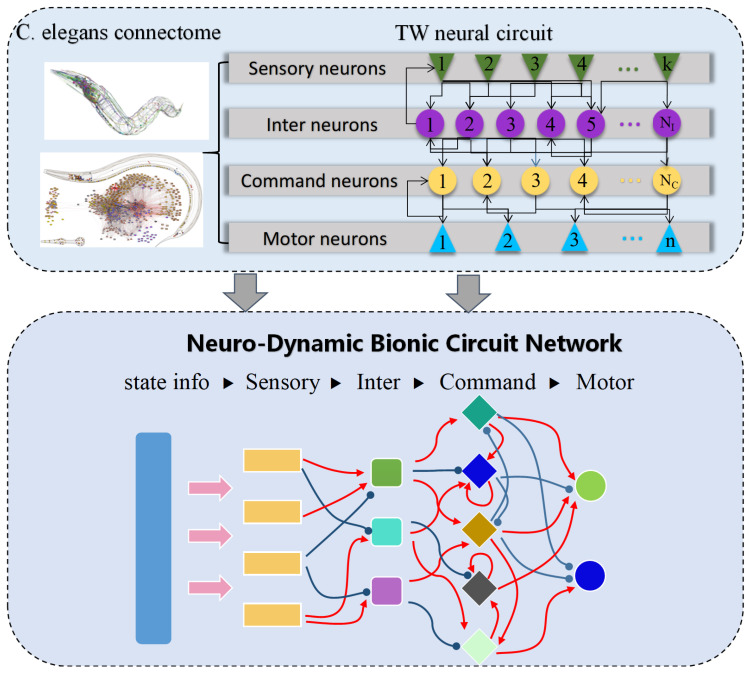
Neuro-dynamic bionic control network.

As illustrated in Figure 2, which corresponds to the low-level controller block in Figure 1, NDBCNet abstracts the C. elegans connectome into four functional layers: sensory, inter, command, and motor neurons. State information is received by sensory neurons, integrated by interneurons, regulated by Command Neurons, and converted into motor actions. Excitatory synapses ($w_{ij}>0$) promote activity, while inhibitory synapses ($w_{ij}<0$) suppress it, yielding a biologically motivated sparse topology that reduces parameters while preserving control diversity.

Each neuron *i* evolves according to membrane potential dynamics:

(15) $$C_{m}\frac{dV_{i}}{dt}=-g_{l}\left(V_{i}-V_{\mathrm{leak}}\right)+\sum_{j\in N_{i}}w_{ij}\sigma \left(V_{j}\right)\left(E_{ij}-V_{i}\right),$$

where $C_{m}$ is membrane capacitance, $g_{l}$ is leak conductance, $V_{\mathrm{leak}}$ is leakage potential, and $E_{ij}$ is the synaptic reversal potential. This formulation enables adaptive temporal processing beyond fixed-step ANN updates.

To ensure stable training and gradient propagation, the dynamics are integrated using the semi-implicit Euler method:

(16) $$V_{i}\left(t+\Delta t\right)=\frac{V_{i}\left(t\right)C_{m}/\Delta t+g_{l}V_{\mathrm{leak}}+\sum_{j}w_{ij}\sigma \left(V_{j}\right)E_{ij}}{C_{m}/\Delta t+g_{l}+\sum_{j}w_{ij}\sigma \left(V_{j}\right)}.$$

This update guarantees bounded local sensitivity and avoids vanishing or exploding gradients during backpropagation.We integrate the continuous-time neuro-dynamics with a semi-implicit Euler scheme and make the integration step δt explicit. To balance stability and responsiveness across tasks, we adopt a bounded, model-aware rule:

(17) $$\delta t\;=\;\mathrm{clip}\;\left(\frac{\eta }{\hat{L}+\varepsilon },\;\delta t_{\min},\;\delta t_{\max}\right),$$

where $\hat{L}$ is a lightweight Lipschitz proxy (e.g., a spectral-norm estimate of the dominant recurrent block or a running bound inferred from weight magnitudes), η>0 is a dimensionless scale, and $\delta t_{\min},\delta t_{\max}$ are task-level safety bounds.

To avoid step-induced drift, we optionally use an embedded half-step check: accept the update if $∥z_{t+\delta t}-z_{t}∥\leq \epsilon_{\mathrm{rel}}∥z_{t}∥+\epsilon_{\mathrm{abs}}$; otherwise sub-step with $\delta t\leftarrow \frac{1}{2}\delta t$ up to a small cap $K_{\max}$. This keeps numerical error controlled while preserving the rhythmic execution required by hierarchical timing.

The effective response time constant of neuron *i* is as follows:

(18) $$\tau_{\mathrm{sys}}^{\left(i\right)}=\frac{1}{\frac{1}{\tau_{i}}+C_{m}\sum_{j}w_{ij}\sigma \left(V_{j}\right)},$$

where smaller values indicate faster responses. This property allows the low-level controller to quickly adapt to high-level subgoal updates.

For deployment, we derive a closed-form approximation to the ODE solution, which significantly accelerates inference without retraining:

(19) $$V\left(t\right)\approx \left(V_{0}-A\right)\exp\;\left(-\left(\frac{w}{\tau }+f\left(I\left(t\right)\right)\right)t\right)+A,$$

with bounded error relative to the exact solution. This efficiency makes NDBCNet well suited for real-time low-level control in hierarchical RL.

NDBCNet provides a compact, interpretable, and dynamically responsive architecture for low-level control, combining biologically motivated sparsity with continuous-time modeling to improve robustness, adaptability, and computational efficiency.

While NCP/LTC integrate all neuron-state evolution into a single continuous-time recurrence, our NDBCNet introduces a modular neuro-dynamic block with two coupled pathways: an input-driven pathway for exogenous signals and a memory pathway for internal state evolution. This modularization enables explicit conditioning of the low-level controller on task variables $\left(g,{\tilde{\Delta t}}_{\mathrm{rem}}\right)$, promotes sparse connectivity for efficiency and interpretability, and provides a clean numerical interface for stable time stepping under fixed execution windows.

For numerical integration, we adopt the semi-implicit Euler scheme to discretize the continuous-time dynamics $\dot{x}=f\left(x,u\right)$ with a dissipative linear term −Kx (where K⪰0) and a bounded nonlinearity ϕ(·). The resulting update reads

(20) $$x_{t+1}\;=\;{\left(I\;+\;\eta K\right)}^{-1}\;\left(x_{t}\;+\;\eta \;\phi \;\left(Wu_{t}\;+\;Ux_{t}\;+\;b\right)\right),$$

where η>0 is the step size. Compared with the explicit Euler form, (20) improves stability by implicitly damping the linear part via ${\left(I+\eta K\right)}^{-1}$ while keeping a simple explicit evaluation of the nonlinear drive.

Under standard bounded-input and Lipschitz assumptions on ϕ (i.e., $∥\phi \left(z\right)∥\leq c_{0}+L∥z∥$) and $K⪰\lambda_{\min}I$ with $\lambda_{\min}\geq 0$, the update (20) admits a bounded-input–bounded-state (BIBS) inequality:

(21) $$∥x_{t+1}∥\;\leq \;\rho \;∥x_{t}∥\;+\;c\;∥u_{t}{∥\;+\;d,\;\rho \;=\;∥\left(I+\eta K\right)}^{-1}∥\;\left(1\;+\;\eta L∥U∥\right),$$

where c,d are finite constants determined by $\eta ,W,b,c_{0}$. By choosing η and the block structure so that ρ<1 (e.g., diagonal or block-diagonal *K* with $\eta \lambda_{\min}$ sufficiently large), (21) ensures asymptotic boundedness of the state in the presence of bounded inputs, mitigating divergence and explaining our empirical robustness under fixed time windows. In practice, we instantiate *K* as diagonal or small block-diagonal so that ${\left(I+\eta K\right)}^{-1}$ reduces to elementwise (or tiny-block) scaling, preserving low compute and latency while retaining the stability benefits of the semi-implicit step.

### 4.4. Low-Level Policy Generation and Optimization

In the proposed framework, the low-level controller is implemented under the SAC algorithm, with its policy network $\pi_{\theta }$ instantiated by the NDBCNet. This design enables the policy to leverage continuous-time neural dynamics for robust and interpretable control.

NDBCNet maps environment states $s_{t}$ and sub-goals $g_{t}$ to continuous actions via membrane potential dynamics. The potentials of intermediate neurons are aggregated as

(22) $$y_{t}=\sum_{i=1}^{N}\alpha_{i}x_{i}\left(t\right),$$

where $x_{i}\left(t\right)$ is the membrane potential of neuron *i* and $\alpha_{i}$ is the output projection weight. The aggregated signal defines the parameters of a Gaussian policy:

(23) $$a_{t}∼\pi_{\theta }\left(a_{t}|s_{t},g_{t}\right)=N\left(\mu \left(s_{t},g_{t}\right),\sigma {\left(s_{t},g_{t}\right)}^{2}\right),$$

with actions sampled using the reparameterization trick for stable gradients:

(24) $$a_{t}=\tanh\;\left(\mu_{\theta }\left(s_{t},g_{t}\right)+\sigma_{\theta }\left(s_{t},g_{t}\right)\cdot \epsilon \right),\;\epsilon ∼N\left(0,1\right).$$

The low-level policy is trained with entropy-regularized SAC, encouraging exploration while stabilizing updates. In practice, the NDBCNet-based controller follows the standard SAC objective, with the aggregated output $y_{t}$ providing the Gaussian parameters (μ,σ).

Compared with conventional multilayer perceptrons, NDBCNet offers: (i) superior temporal modeling through continuous-time dynamics, (ii) enhanced interpretability from sparse and modular architecture, (iii) improved stability via recurrent structure and bounded gradient propagation, and (iv) high responsiveness within each subgoal execution window τ. These properties improve robustness against noise, accelerate convergence, and enhance low-level control precision in dynamic, long-horizon tasks.

### 4.5. Strategy Optimization and Training Procedure

In TBC-HRL, the high-level policy $\pi_{1}$ and the low-level policy $\pi_{0}$ (parameterized by NDBCNet) are independently optimized under the SAC algorithm within a SMDP formulation. The high-level policy $\pi_{1}$ generates subgoals $g_{t}$ together with time budgets Δt, which define both the spatial target and the execution horizon. The low-level policy $\pi_{0}$ conditions on the current state $s_{t}$, the assigned subgoal $g_{t}$, and the remaining time $\Delta t_{\mathrm{rem}}$, enabling time-aware goal-conditioned control.

Low-level training: After receiving a subgoal $g_{t}$ and time budget Δt, the low-level policy interacts with the environment for up to Δt steps (or until termination), decrementing $\Delta t_{\mathrm{rem}}$ at each step. Each transition is stored in buffer $D_{0}$ as

$$(s_{t},g_{t},\Delta t_{\mathrm{rem}},a_{t},r_{t},s_{t+1},\Delta t_{\mathrm{rem}}-1),$$

which provides a time-aware representation for experience replay and hindsight relabeling. This allows the low-level controller to learn not only how to reach subgoals but also how to allocate actions within a fixed temporal budget.

High-level training: When a subgoal finishes execution (either successfully or by exhausting $\Delta t_{\mathrm{rem}}$), the high-level reward $r_{1}$ is computed as the cumulative environment reward over the interval, optionally with a completion bonus or penalty. The high-level transition is stored as

$$(s_{t},g_{t},\tau ,r_{1},s_{t+\tau },\mathrm{done}),$$

where τ is the actual number of low-level steps, and discounting $\gamma^{\tau }$ is applied in buffer $D_{1}$. To further improve sample efficiency, Hindsight Goal Relabeling (HGR) is employed: if the intended subgoal is not reached, the final achieved state is substituted as a new subgoal while keeping Δt unchanged, and the corresponding rewards are recalculated. This densifies reward signals and mitigates the non-stationarity of high-level transitions.

With this scheme, the low-level controller learns precise, temporally constrained behaviors, while the high-level policy focuses on task decomposition and subgoal scheduling. Together, they improve exploration efficiency, robustness, and temporal coordination in long-horizon tasks. The complete training process is summarized in Algorithm 1.
**Algorithm 1** TBC-HRL: Strategy Optimization and Training Procedure 1:**Init:** high-level SAC $\pi_{1}$, low-level SAC $\pi_{0}$ (NDBCNet), twin critics $Q_{1,1},Q_{1,2}$ and $Q_{0,1},Q_{0,2}$, target critics $Q_{1,i}^{\mathrm{tgt}},Q_{0,i}^{\mathrm{tgt}}$, replay buffers $D_{1},D_{0}$, optimizers with LR $\left(\eta_{1},\eta_{0}\right)$, discounts $\left(\gamma ,\gamma^{\tau }\right)$, temperatures $\left(\alpha_{1},\alpha_{0}\right)$, Polyak factor $\tau_{\mathrm{polyak}}$, batch sizes $\left(B_{1},B_{0}\right)$, update steps $\left(K_{1},K_{0}\right)$ 2:**for** each episode **do** 3:   Reset env; get $s_{t}$ 4:   **while** episode not terminated **do** 5:     Sample high-level action $\left(g,\Delta t\right)∼\pi_{1}\left(\cdot |s_{t}\right)$; set $\Delta t_{\mathrm{rem}}\;\leftarrow \;\Delta t$, τ←0, $R_{\mathrm{sum}}\;\leftarrow \;0$, $s_{\mathrm{start}}\;\leftarrow \;s_{t}$ 6:     **for** k=0 to Δt−1 **do** 7:        Sample $a∼\pi_{0}\left(\cdot |s_{t},g,\Delta t_{\mathrm{rem}}\right)$ 8:        Execute *a*; observe $s_{t+1}$, reward $r_{0}$, done 9:        Store $\left(s_{t},g,\Delta t_{\mathrm{rem}},a,r_{0},s_{t+1},\Delta t_{\mathrm{rem}}-1\right)$ in $D_{0}$10:        $R_{\mathrm{sum}}\leftarrow R_{\mathrm{sum}}+r_{0}$;   $\Delta t_{\mathrm{rem}}\leftarrow \Delta t_{\mathrm{rem}}-1$;   τ←τ+111:        **// low-level updates**12:        **for** u=1 to $K_{0}$ **do**13:          Sample batch ${\left\{\left(s,g,\delta ,a,r,s^{\prime },\delta^{\prime }\right)\right\}}_{B_{0}}\;∼\;D_{0}$;   sample $a^{\prime }\;∼\;\pi_{0}\left(\cdot |s^{\prime },g,\max\left(\delta^{\prime },0\right)\right)$14:          $y_{0}\leftarrow r+\gamma \left(\min_{i}Q_{0,i}^{\mathrm{tgt}}\left(s^{\prime },g,\max\left(\delta^{\prime },0\right),a^{\prime }\right)-\alpha_{0}\log\pi_{0}\left(a^{\prime }|s^{\prime },g,\max\left(\delta^{\prime },0\right)\right)\right)$15:          Update $Q_{0,i}$ towards $y_{0}$;   update $\pi_{0}$ with respect to $\alpha_{0}\log\pi_{0}-\min_{i}Q_{0,i}$16:          Soft-update $Q_{0,i}^{\mathrm{tgt}}\leftarrow \tau_{\mathrm{polyak}}Q_{0,i}+\left(1-\tau_{\mathrm{polyak}}\right)Q_{0,i}^{\mathrm{tgt}}$17:          (optional) adjust $\alpha_{0}$ by target entropy18:        **end for**19:        t←t+1;   $s_{t}\leftarrow s_{t+1}$20:        **if** done **or** GoalReached($s_{t},g$) **or** $\Delta t_{\mathrm{rem}}=0$ **then**21:          **break**22:        **end if**23:     **end for**24:     **// aggregate and store high-level SMDP transition**25:     $r_{1}\leftarrow R_{\mathrm{sum}}$ (plus success bonus/penalty)26:     Store $\left(s_{\mathrm{start}},g,\tau ,r_{1},s_{t},\mathrm{done}\right)$ in $D_{1}$27:     **if not** GoalReached($s_{t},g$) **then**28:        $g^{\prime }\leftarrow s_{t}$ Hindsight Goal Relabeling (HGR)29:        recompute $r_{1}^{\prime }$30:        Store $\left(s_{\mathrm{start}},g^{\prime },\tau ,r_{1}^{\prime },s_{t},\mathrm{done}\right)$ in $D_{1}$31:     **end if**32:     **// high-level updates**33:     **for** u=1 to $K_{1}$ **do**34:        Sample batch ${\left\{\left(s,g,\tau ,r_{1},s^{+},d\right)\right\}}_{B_{1}}\;∼\;D_{1}$;   sample $\left(g^{+},\Delta t^{+}\right)\;∼\;\pi_{1}\left(\cdot |s^{+}\right)$35:        $y_{1}\leftarrow r_{1}+\gamma^{\tau }\;\left(\min_{i}Q_{1,i}^{\mathrm{tgt}}\left(s^{+},g^{+},\Delta t^{+}\right)-\alpha_{1}\log\pi_{1}\left(g^{+},\Delta t^{+}|s^{+}\right)\right)$36:        Update $Q_{1,i}$ towards $y_{1}$;   update $\pi_{1}$ with respect to $\alpha_{1}\log\pi_{1}-\min_{i}Q_{1,i}$37:        Soft-update $Q_{1,i}^{\mathrm{tgt}}\leftarrow \tau_{\mathrm{polyak}}Q_{1,i}+\left(1-\tau_{\mathrm{polyak}}\right)Q_{1,i}^{\mathrm{tgt}}$38:        (optional) adjust $\alpha_{1}$ by target entropy39:     **end for**40:   **end while**41:**end for**42:**return** trained $\pi_{1}\left(\theta_{1}\right)$, $\pi_{0}\left(\theta_{0}\right)$

Per low-level control step, the semi-implicit Euler integrator uses *K* sub-steps ($K\leq K_{\max}$), each performing one linear pass over active synapses. The time complexity is O(E) with *E* the number of effective connections (fan-out and recurrent links), and memory is O(E+N) with *N* neurons. Because *K* is capped and δt is clipped by (17), the worst-case overhead remains linear and predictable. We keep the implementation vectorizable and single-precision to reduce latency; environment-specific bounds $\left(\delta t_{\min},\delta t_{\max}\right)$ are listed in Appendix A Table A2.

## 5. Experiments

### 5.1. Experimental Environments

**Figure 3:**
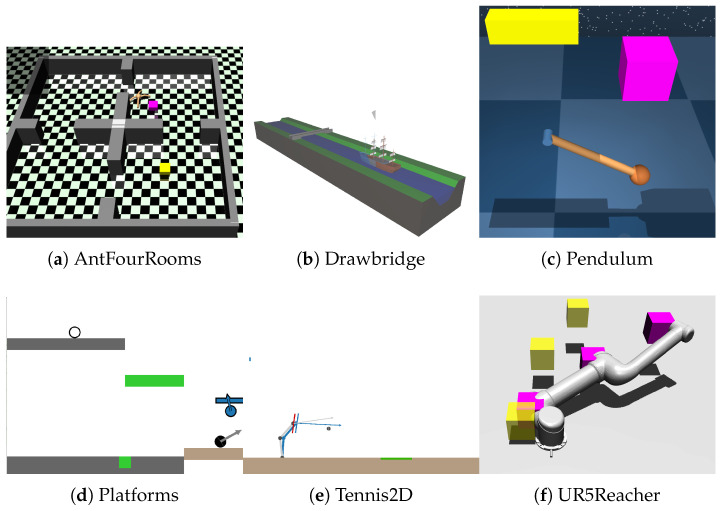
Six simulation environments used in the experiments.

To comprehensively evaluate the proposed TBC-HRL algorithm, we conducted experiments in six representative simulation environments that differ in task difficulty, dynamics, and control requirements (see Figure 3). The training steps, state/action dimensions, and key characteristics of these environments are summarized in Table 1. The selected environments cover a broad spectrum of tasks, including navigation, manipulation, balance control, and dynamic interaction, providing a solid basis for assessing performance under long-horizon dependencies and sparse rewards.

- AntFourRooms:A quadrupedal robot navigates through a four-room maze from a start point to a designated goal room. The environment contains narrow passages and obstacles, emphasizing long-horizon planning and obstacle avoidance.
- Drawbridge: A timing-control scenario where the agent must operate a drawbridge to allow ships to pass safely. The task highlights temporal coordination and proactive anticipation in dynamic environments.
- Pendulum: A classic control problem requiring the pendulum to be swung upright and stabilized at the top. Its nonlinear dynamics and continuous action space demand precise force application and balance maintenance.
- Platforms: A side-scrolling style task where the agent must trigger moving platforms at the correct moment to reach the target. Delayed action effects and sparse rewards make it a benchmark for temporal reasoning and credit assignment.
- Tennis2D: A robotic arm must strike a ball so that it lands in a target zone. Success requires accurate timing under high stochasticity and frequent contacts, with minimal latency in control.
- UR5Reacher: An industrial robotic arm control task involving reaching multiple targets while avoiding collisions. It evaluates accuracy, path efficiency, and energy minimization in high-degree-of-freedom systems.

Across all environments, we report success rate, sample efficiency (measured as the number of training steps required to reach a performance threshold), and policy stability (variance) as the main evaluation metrics. Importantly, the characteristics of these environments align with the core contributions of TBC-HRL: long horizons and sparse rewards emphasize the benefits of timed subgoal scheduling, while dynamic perturbations and high-dimensional control highlight the responsiveness and interpretability advantages of NDBCNet. Complete NDBCNet configurations and per-algorithm hyperparameters are provided in Appendix A Table A1 and Table A2.

To assess whether observed improvements are statistically significant, we compute two-sided significance tests and confidence intervals using the runs already collected (no additional training). Unless noted otherwise, we aggregate per-environment scores across independent seeds, and within each seed across evaluation episodes.

For each environment and method, we report the mean ± standard deviation and add 95% bootstrap confidence intervals (10,000 resamples) over seed means. (When fewer than three seeds are available, we instead bootstrap episode returns per seed with a block size equal to the evaluation window and combine across seeds (paired bootstrap).) For pairwise comparisons against the strongest non-ablated baseline in the same environment, we run a two-sided Welch’s *t*-test on the seed means (or a paired bootstrap test when normality is doubtful), and control the family-wise error rate across multiple environments using Holm–Bonferroni at α=0.05. We also report an effect size (Cliff’s δ) in the supplement. We mark significant improvements over the best baseline with * (p<0.05) and ** (p<0.01) after Holm adjustment; non-significant differences are left unmarked.

### 5.2. Experimental Results

**Figure 4:**
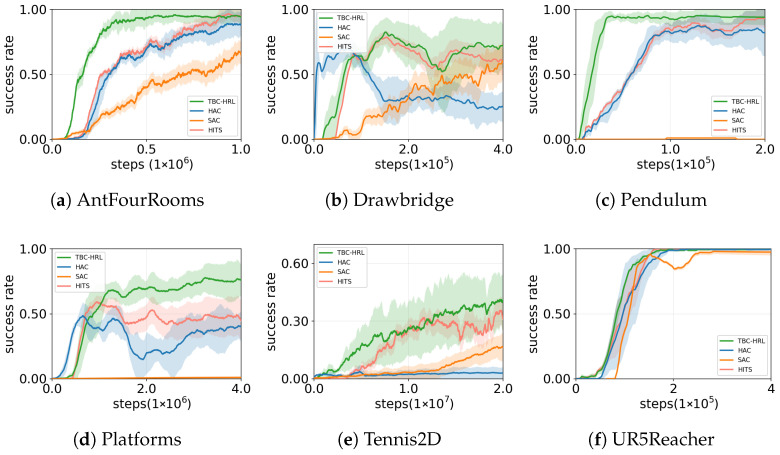
Main experiment success rates across the six environments.

We compare four methods across six environments (Figure 4, Table 2): SAC, HAC, HITS, and TBC-HRL. SAC is a single-layer baseline; HAC is a two-level variant that does not include timed subgoal scheduling or NDBCNet; HITS is a time-aware hierarchical reinforcement learning baseline in which a high-level policy proposes subgoals and a low-level controller executes them; TBC-HRL augments the hierarchical framework with a timed subgoal mechanism and NDBCNet. All methods share identical state/action spaces, reward functions, and training budgets. In Table 2, convergence gain denotes the reduction in training steps for TBC-HRL to reach a target threshold relative to the strongest baseline (the best among SAC/HAC/HITS). Shaded bands indicate 95% bootstrap confidence intervals, and final-epoch markers report significance against the strongest baseline. To ensure fair comparison, we match hyperparameter tuning and budgets, report results over 10 random seeds, aggregate by final-epoch mean ± SD, use a common success threshold for convergence, and present 95% bootstrap confidence intervals.

Time-critical environments (Drawbridge, Platforms, Tennis2D). In tasks requiring clear temporal rhythm and phase progression, TBC-HRL consistently converges fastest and attains the highest final success rates. Overall, TBC-HRL typically reaches the target about 0.4–0.8 M steps earlier than the strongest baseline with smoother learning curves. For example, on Platforms, TBC-HRL reaches about 72% success versus roughly 43% for HITS. On Tennis2D, TBC-HRL reaches about 38% versus about 24% for HITS, yet none of the methods achieve stable convergence within the budget, so convergence gain is not reported.

Precision control and dynamic responsiveness (AntFourRooms, UR5Reacher, Pendulum). In tasks emphasizing fine control and rapid response, TBC-HRL again achieves the best final success and more stable convergence. On AntFourRooms, TBC-HRL is about 90% compared to roughly 84% for HITS; on UR5Reacher, 97.6% versus 96.6%, with lower variance and smoother curves. On Pendulum, all methods quickly reach high performance, leaving limited headroom for further gains.

In summary, across all six environments TBC-HRL attains the best final success rates. It shows clear sample-efficiency advantages in time-critical tasks and more stable high performance in precision-control tasks. The combination of timed subgoals (stabilizing inter-level coordination and long-horizon credit assignment) and NDBCNet (enhancing low-level precision and adaptability) explains the observed profile: faster learning, lower variance, and higher asymptotic success.

**Figure 5:**
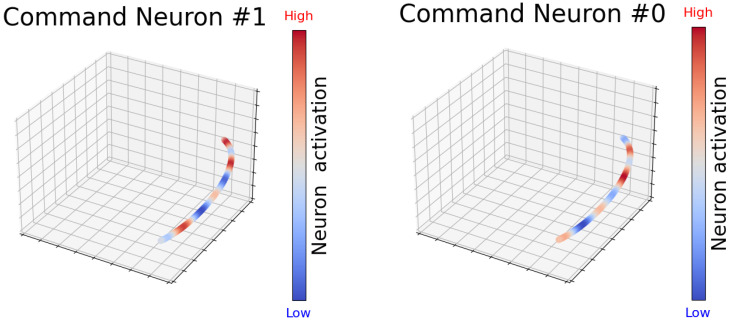
UR5 command-neuron activations.

Figure 5 illustrates the activation dynamics of different Command Neurons for the third-joint motion in the UR5Reacher environment. The visualizations transition from low (blue) to high (red) along the spatial trajectory, reflecting how TBC-HRL processes time-series signals during motion execution.

In UR5Reacher, Command Neuron #0 remains highly active in specific spatial regions, indicating sensitivity to particular joint configurations or orientations, whereas Command Neuron #1 dominates in other regions. This division of labor suggests that NDBCNet promotes functional specialization among neurons to capture distinct motion features, thereby improving control accuracy and coordination.

To provide quantitative support consistent with these patterns, we evaluate four complementary measures on held-out trajectories. Lesion experiments show that removing the top-5 most active neurons yields a relative performance drop of 12.8±2.3%. The average Pearson correlation between neuron activities and task features (e.g., speed, altitude change, and energy consumption) is 0.47±0.06. Finally, the mutual information between neuron activations and subgoal phases is 0.36 bits on average (0.52 bits at the 90th percentile). These results align with the spatial–temporal activation layouts in Figure 5, indicating phase-specific responses within the subgoal window and structured internal dynamics that help explain the model’s decision process in complex control tasks.

### 5.3. Ablation Study

For ablations (TS-off and NDBCNet-off), we match the total number of parameters within ±0.5% by width adjustments or zero-padding and exactly match the training budgets (total environment steps, gradient updates, batch sizes, replay ratios, and evaluation frequency). Optimizer schedules and entropy-temperature targets are kept fixed across variants.

**Figure 6:**
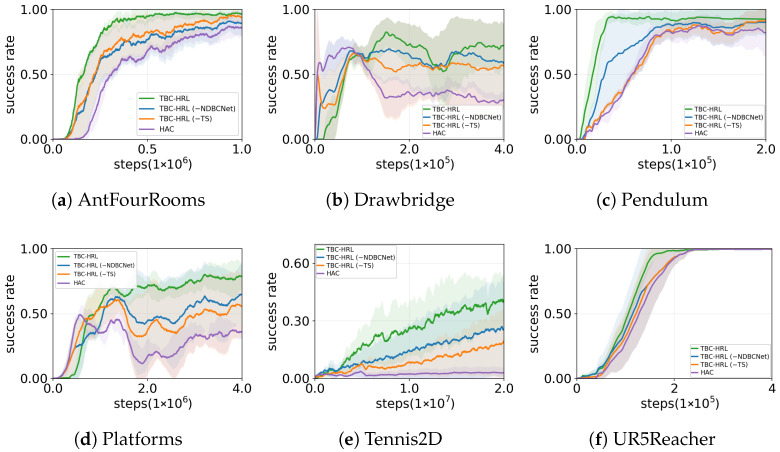
Ablation study: success rate curves across six environments.

We compare four configurations (Figure 6 and Table 3): (1) full TBC-HRL with both NDBCNet and timed subgoals (TS), (2) TBC-HRL(-NDBCNet) where the low-level policy is replaced by an MLP but TS is retained, (3) TBC-HRL(-TS) where NDBCNet is preserved but TS is removed, and (4) HAC without either component.

Overall, TBC-HRL consistently achieves the best success rates, faster convergence, and lower variance. Removing either TS or NDBCNet degrades performance, while HAC is the weakest and most unstable, confirming that both components are necessary and complementary.

Task-level analysis shows distinct roles. In spatially complex or high-DoF tasks such as AntFourRooms and UR5Reacher, NDBCNet is critical: TBC-HRL achieves about 90% in AntFourRooms, compared to 71% without NDBCNet and 75% for HAC. In timing-sensitive environments such as Drawbridge, Platforms, and Tennis2D, TS is more important:; for example, in Drawbridge success drops from 71% (full) to 57% without TS and 36% for HAC. Pendulum is relatively simple: all methods exceed 80%, with TBC-HRL showing slightly faster and more stable convergence.

Mechanistically, TS stabilizes inter-level coordination by assigning fixed execution durations τ, thereby improving temporal credit assignment in timing-critical settings. NDBCNet, with its sparse connectivity and continuous-time dynamics, more effectively captures nonlinearities and delays, leading to enhanced precision in high-dimensional control. The synergy between these modules accounts for the improved convergence speed, stability, and asymptotic performance observed in TBC-HRL, while the systematic drops under each ablation underscore their complementary contributions. Quantitatively, removing TS reduces the average success rate by about 14% in timing-sensitive environments (Drawbridge, Platforms, Tennis2D), while removing NDBCNet lowers performance by roughly 11% in spatially complex tasks (AntFourRooms, UR5Reacher), further confirming their distinct yet complementary roles.

## 6. Discussion

This work introduces TBC-HRL, which integrates timed subgoal scheduling with an NDBCNet for low-level control. As demonstrated in Figure 4 and confirmed by the ablation study in Figure 6, the full framework achieves higher asymptotic success, faster convergence, and lower across-seed variability compared with HAC and single-component variants.

Environment-specific analyses reveal task-dependent patterns: spatially complex or high-DoF settings (AntFourRooms, UR5Reacher) benefit more from NDBCNet, while timing- or phase-critical tasks (Drawbridge, Tennis2D, Platforms) gain more from TS. In the simple Pendulum task, all methods perform comparably, but TBC-HRL still converges slightly faster and more stably. Mechanistically, TS enforces a fixed execution duration τ, which mitigates inter-level chattering and improves temporal credit assignment, whereas NDBCNet introduces sparse, continuous-time dynamics that better capture nonlinearities and delays.

Importantly, the synergy of TS and NDBCNet consistently enhances convergence speed, stability, and final performance across diverse environments. Quantitatively, removing TS lowers average success rates by about 14% in timing-sensitive tasks, while removing NDBCNet reduces performance by roughly 11% in spatially complex settings. Neuron-level visualizations further highlight functional specialization within controllers, linking these gains to improved interpretability. Beyond empirical improvements, these findings suggest that incorporating biologically inspired temporal abstraction and neural dynamics represents a new design paradigm for stable and interpretable hierarchical reinforcement learning.

Our step-size policy (17) bounds the number of sub-steps and keeps the per-cycle work linear in the active synapses, which is amenable to real-time loops on resource-constrained platforms. While we defer full hardware profiling to future work, the present design (single-precision, vectorizable kernels, bounded $K_{\max}$) targets predictable latency budgets typical of embedded control.

## 7. Conclusions

In summary, TBC-HRL achieves consistent improvements in convergence speed, stability, and final performance across six benchmark environments by integrating timed subgoal scheduling with the NDBCNet. The results demonstrate that TS reduces inter-level chattering and strengthens temporal credit assignment in timing-sensitive tasks, while NDBCNet provides sparse, continuous-time dynamics that enhance precision and robustness in spatially complex or high-dimensional settings. Neuron-level visualizations further reveal functional specialization within the learned controllers, offering improved interpretability and linking the observed performance gains to biologically inspired design principles.

Despite these contributions, this study has several limitations, including the use of a fixed τ and the focus on simulated environments. Future research will explore adaptive or learned scheduling strategies, expand comparisons to broader baselines including model-based and hierarchical approaches, and investigate robustness under non-stationary and transfer settings. In addition, sim-to-real validation and causal interpretability probes will be pursued to further substantiate the biological inspiration and practical applicability of the proposed framework.

## Figures and Tables

**Table 1:** Experimental environments and training configurations.

Environment	Training Steps	State/Action Dim.	Key Characteristics
AntFourRooms	1.0 M	s=111, a=8	Four-room maze, sparse rewards, long-horizon planning
Drawbridge	0.4 M	s=85, a=1	Dynamic obstacle, timing-critical actions
Pendulum	0.2 M	s=3, a=1	Classic control, simple dynamics
Platforms	4.0 M	s=75, a=1	Multi-stage navigation, sparse rewards
Tennis2D	20 M	s=30, a=3	High stochasticity, frequent contacts
UR5Reacher	0.4 M	s=48, a=3	Robotic arm reaching, high precision

**Table 2:** Success rate (%) and convergence gain of SAC, HAC, HITS, and TBC-HRL across six environments.

Environment	SAC Success (%)	HAC Success (%)	HITS Success (%)	TBC-HRL Success (%)	Convergence Gain (M Env Steps; vs. Best Baseline)
AntFourRooms	65.2±7.1	74.6±10.8	84.3±9.5	90.1±8.7 *	+0.5 M
Drawbridge	51.3±10.7	36.2±9.3	58.4±12.0	70.8±12.1 *	+0.4 M
Pendulum	4.8±1.2	80.6±11.8	88.9±9.0	90.7±8.2 *	+0.05 M
Platforms	2.2±0.9	34.7±11.2	43.2±12.5	72.4±16.6 *	+0.8 M
Tennis2D	14.5±5.1	4.2±4.1	24.3±6.0	37.6±9.8 *	N/A ^†^
UR5Reacher	90.9±2.1	95.8±3.6	96.6±2.5	97.6±2.1 *	+0.01 M

Means ± SD over 10 seeds; 95% bootstrap CIs are reported in Appendix A Table A1 and Table A2. Pairwise tests vs. the strongest non-ablated baseline use Welch’s *t*-test with Holm–Bonferroni (α=0.05). ^†^ In Tennis2D, no method reached stable convergence within the budget; convergence gain not reported. *: significant vs. strongest baseline (p<0.05; Welch + Holm–Bonferroni).

**Table 3:** Ablation study: average success rates (%) across six environments (means ± SD over 10 seeds).

Environment	HAC	TBC-HRL (-NDBCNet)	TBC-HRL (-TS)	TBC-HRL
AntFourRooms	74.6±10.8	71.5±8.7	82.4±9.2	** 90.1±8.7 **
Drawbridge	36.2±9.3	63.3±15.1	57.4±18.8	** 70.8±12.1 **
Pendulum	80.6±11.8	85.0±7.5	83.1±8.3	** 90.7±8.2 **
Platforms	34.7±11.2	52.6±14.9	61.1±15.1	** 72.4±16.6 **
Tennis2D	4.2±4.1	13.7±9.3	26.9±15.0	** 37.6±9.8 **
UR5Reacher	95.8±3.6	95.3±2.7	96.0±2.5	** 97.6±2.1 **

Ablations match total parameters (within ±0.5%) and training budgets (env steps, updates, batch sizes, replay ratios, eval frequency). Pairwise tests vs. TBC-HRL use Welch’s *t*-test with Holm–Bonferroni (α=0.05).

## Data Availability

The original contributions presented in this study are included in the article. Further inquiries can be directed to the corresponding author.

## Appendix A

**Table A1:** NDBCNet hyperparameters per environment.

Environment	Inter Neurons	Command Neurons	Motor Neurons	Sensory Fanout	Inter Fanout	Recurrent Command Synapses	Motor Fanin
AntFourRooms	12	10	8	5	8	2	6
UR5Reacher	6	4	3	4	5	2	5
Drawbridge	6	6	1	3	4	3	4
Tennis2D	8	6	3	4	6	4	6
Pendulum	6	6	1	3	4	1	4
Platforms	6	6	1	4	5	3	5

*Notes.* “Motor neurons” typically matches the action dimension of each environment. Values shown here are the per-environment overrides used in our main experiments.

**Table A2:** Key hyperparameters per algorithm across environments.

Algorithm	Env	LR	Batch	Alpha (L0/L1)	Polyak τ	Hindsight	$\Delta t_{\max}$	Max *n* Actions (HL2)	κ (Δt Scaling)
SAC	AntFourRooms	2.404 × 10^−3^	1024	target/—	3.290 × 10^−3^	3	—	—	—
	Drawbridge	9.602 × 10^−3^	256	0.6836/—	9.823 × 10^−3^	3	—	—	—
	Pendulum	8.955 × 10^−4^	256	target/—	2.650 × 10^−4^	3	—	—	—
	Platforms	3.752 × 10^−4^	1024	0.00208/—	0.00780	3	—	—	—
	Tennis2D	6.355 × 10^−4^	1024	target/—	1.056 × 10^−4^	1	—	—	—
	UR5Reacher	2.167 × 10^−3^	1024	0.005156/—	0.06666	3	—	—	—
HiTS	AntFourRooms	9.289 × 10^−4^	1024	0.00261/1.1267	1.086 × 10^−3^	3	^−1^	22	—
	Drawbridge	7.228 × 10^−5^	256	0.05413/0.02071	0.3144	3	^−1^	5	—
	Pendulum	6.441 × 10^−3^	256	target/2.5253	0.01259	3	^−1^	22	—
	Platforms	1.940 × 10^−4^	512	0.00419/1.1173	0.02142	3	^−1^	10	—
	Tennis2D	5.680 × 10^−4^	1024	0.03605/target	8.910 × 10^−5^	3	^−1^	8	—
	UR5Reacher	4.968 × 10^−4^	1024	0.000532/target	0.02726	3	^−1^	24	—
TBC–HRL	AntFourRooms	9.753 × 10^−4^	1024	0.00261/1.1267	1.200 × 10^−3^	3	^−1^	22	0.95
	Drawbridge	7.589 × 10^−5^	256	0.05413/0.02071	0.3460	3	−1	5	0.85
	Pendulum	6.763 × 10^−3^	256	target/2.5253	0.01390	3	−1	22	1.10
	Platforms	2.037 × 10^−4^	512	0.00419/1.1173	0.02360	3	−1	10	1.00
	Tennis2D	5.964 × 10^−4^	1024	0.03605/target	9.900 × 10^−5^	3	−1	8	0.90
	UR5Reacher	5.216 × 10^−4^	1024	0.000532/target	0.03000	3	−1	24	1.05
HAC	AntFourRooms	1.652 × 10^−3^	1024	0.04873/target	1.710 × 10^−3^	3	—	17	—
	Drawbridge	2.147 × 10^−4^	1024	0.001009/target	0.2031	3	—	5	—
	Pendulum	1.707 × 10^−3^	256	9.593 × 10^−5^/0.3715	0.01698	3	—	27	—
	Platforms	1.436 × 10^−4^	1024	0.00460/1.6896	0.01244	3	—	10	—
	Tennis2D	6.913 × 10^−5^	1024	2.905 × 10^−4^/target	6.278 × 10^−4^	3	—	8	—
	UR5Reacher	4.332 × 10^−3^	1024	0.02822/0.4880	0.01199	3	—	24	—

*Notes.* “target” denotes automatic temperature via target entropy; a— in L1 indicates a single-level method. $\Delta t_{\max}$ caps the high-level execution window when used; κ is TBC-HRL’s temporal scaling that maps reachability to the prescribed Δt.

## References

[1] Zhu K., Zhang T. (2021). Deep reinforcement learning based mobile robot navigation: A review. Tsinghua Sci. Technol..

[2] Hu Y., Wang S., Xie Y., Zheng S., Shi P., Rudas I., Cheng X. (2025). Deep reinforcement learning-based mapless navigation for mobile robot in unknown environment with local optima. IEEE Robot. Autom. Lett..

[3] Hu J., Niu H., Carrasco J., Lennox B., Arvin F. (2020). Voronoi-based multi-robot autonomous exploration in unknown environments via deep reinforcement learning. IEEE Trans. Veh. Technol..

[4] Ibarz J., Tan J., Finn C., Kalakrishnan M., Pastor P., Levine S. (2021). How to train your robot with deep reinforcement learning: Lessons we have learned. Int. J. Robot. Res..

[5] Wang X., Wang S., Liang X., Zhao D., Huang J., Xu X., Dai B., Miao Q. (2024). Deep reinforcement learning: A survey. IEEE Trans. Neural Netw. Learn. Syst..

[6] Pateria S., Subagdja B., Tan A.H., Quek C. (2021). Hierarchical reinforcement learning: A comprehensive survey. ACM Comput. Surv..

[7] Liu C., Zhu F., Liu Q., Fu Y. (2021). Hierarchical reinforcement learning with automatic sub-goal identification. IEEE-CAA J. Autom. Sin..

[8] Yu L.S., Marin A., Hong F., Lin J. Studies on hierarchical reinforcement learning in multi-agent environment. Proceedings of the 2008 IEEE International Conference on Networking, Sensing and Control.

[9] Chai R., Niu H., Carrasco J., Arvin F., Yin H., Lennox B. (2024). Design and experimental validation of deep reinforcement learning-based fast trajectory planning and control for mobile robot in unknown environment. IEEE Trans. Neural Netw. Learn. Syst..

[10] Aradi S. (2022). Survey of deep reinforcement learning for motion planning of autonomous vehicles. IEEE Trans. Intell. Transp. Syst..

[11] Teng S., Chen L., Ai Y., Zhou Y., Xuanyuan Z., Hu X. (2023). Hierarchical interpretable imitation learning for end-to-end autonomous driving. IEEE Trans. Intell. Veh..

[12] Lei K., Guo P., Wang Y., Zhang J., Meng X., Qian L. (2024). Large-scale dynamic scheduling for flexible job-shop with random arrivals of new jobs by hierarchical reinforcement learning. IEEE Trans. Ind. Inf..

[13] Wang X., Garg S., Lin H., Hu J., Kaddoum G., Piran M.J., Hossain M.S. (2022). Toward accurate anomaly detection in industrial Internet of Things using hierarchical federated learning. IEEE Internet Things J..

[14] Liang H., Zhu L., Yu F.R. (2024). Collaborative edge intelligence service provision in blockchain empowered urban rail transit systems. IEEE Internet Things J..

[15] Wei T., Webb B. A bio-inspired reinforcement learning rule to optimise dynamical neural networks for robot control. Proceedings of the 2018 IEEE/RSJ International Conference on Intelligent Robots and Systems (IROS).

[16] Xu K., Li Y., Sun J., Du S., Di X., Yang Y., Li B. (2025). Targets capture by distributed active swarms via bio-inspired reinforcement learning. Sci. China Phys. Mech. Astron..

[17] Gruber R., Schiestl M., Boeckle M., Frohnwieser A., Miller R., Gray R.D., Clayton N.S., Taylor A.H. (2019). New Caledonian crows use mental representations to solve metatool problems. Curr. Biol..

[18] Lechner M., Hasani R., Amini A., Henzinger T.A., Rus D., Grosu R. (2020). Neural circuit policies enabling auditable autonomy. Nat. Mach. Intell..

[19] Ocana F.M., Suryanarayana S.M., Saitoh K., Kardamakis A.A., Capantini L., Robertson B., Grillner S. (2015). The lamprey pallium provides a blueprint of the mammalian motor projections from cortex. Curr. Biol..

[20] Bacon P.-L., Harb J., Precup D. The Option-Critic Architecture. Proceedings of the 31st AAAI Conference on Artificial Intelligence (AAAI).

[21] Vezhnevets A.S., Osindero S., Schaul T., Heess N., Jaderberg M., Silver D., Kavukcuoglu K. FeUdal Networks for Hierarchical Reinforcement Learning. Proceedings of the 34th International Conference on Machine Learning (ICML).

[22] Nachum O., Gu S., Lee H., Levine S. (2018). Data-Efficient Hierarchical Reinforcement Learning. Advances in Neural Information Processing Systems (NeurIPS).

[23] Gürtler N., Büchler D., Martius G. (2021). Hierarchical Reinforcement Learning with Timed Subgoals. Advances in Neural Information Processing Systems (NeurIPS).

[24] Florensa C., Duan Y., Abbeel P. Stochastic Neural Networks for Hierarchical Reinforcement Learning. Proceedings of the International Conference on Learning Representations (ICLR).

[25] Wang R., Li Y., Jin Y. (2020). Spiking Neural Networks: A Survey. IEEE Trans. Neural Netw. Learn. Syst..

[26] Hasani R., Lechner M., Amini A., Liebenwein L., Ray A., Tschaikowski M., Teschl G., Rus D. (2022). Closed-form continuous-time neural networks. Nat. Mach. Intell..

[27] Tylkin P., Harakeh A., Hasani R., Allen R., Siu H.C., Wrafter D., Seyde T., Amini A., Rus D. (2022). Interpretable autonomous flight via compact visualizable neural circuit policies. IEEE Robot. Autom. Lett..

[28] Chahine M., Hasani R., Kao P., Ray A., Shubert R., Lechner M., Amini A., Rus D. (2023). Robust flight navigation out of distribution with liquid neural networks. Sci. Robot..

[29] Du J., Bai Y., Li Y., Geng J., Huang Y., Chen H. (2024). Evolutionary end-to-end autonomous driving model with continuous-time neural networks. IEEE/ASME Trans. Mechatron..

